# RPN2-mediated glycosylation of tetraspanin CD63 regulates breast cancer cell malignancy

**DOI:** 10.1186/1476-4598-13-134

**Published:** 2014-05-31

**Authors:** Naoomi Tominaga, Keitaro Hagiwara, Nobuyoshi Kosaka, Kimi Honma, Hitoshi Nakagama, Takahiro Ochiya

**Affiliations:** 1 Division of Molecular and Cellular Medicine, National Cancer Center Research Institute, Tokyo, Japan; 2 Graduate School of Medicine, The University of Tokyo, Tokyo, Japan; 3 Graduate School of Bioscience and biotechnology, Tokyo Institute of Technology, Tokyo, Japan; 4 Division of Cancer Development System, National Cancer Center Research Institute, Tokyo, Japan

## Abstract

**Background:**

The tetraspanin CD63 is a highly N-glycosylated protein that is known to regulate cancer malignancy. However, the contribution of glycosylation of CD63 to cancer malignancy remains unclear. Previously, we reported that ribophorin II (RPN2), which is part of an N-oligosaccharyle transferase complex, is responsible for drug resistance in breast cancer cells. In this study, we demonstrate that cancer malignancy associated with the glycosylation of CD63 is regulated by RPN2.

**Results:**

Inhibition of RPN2 expression led to a reduction in CD63 glycosylation. In addition, the localization of CD63 was deregulated by knockdown of RPN2. Interestingly, multidrug resistance protein 1 (MDR1) localization was displaced from the cell surface in CD63-silenced cells. CD63 silencing reduced the chemoresistance and invasion ability of malignant breast cancer cells. Furthermore, the enrichment of CD63/MDR1-double positive cells was associated with lymph node metastasis. Taken together, these results indicated that high glycosylation of CD63 by RPN2 is implicated in clinical outcomes in breast cancer patients.

**Conclusions:**

These findings describe a novel and important function of RPN2-mediated CD63 glycosylation, which regulates MDR1 localization and cancer malignancy, including drug resistance and invasion.

## Background

The tetraspanin family is a group of cell surface proteins that are characterized by four transmembrane domains [1]. It is well known that tetraspanin proteins regulate several types of physiological properties, including cell morphology, motility, invasion, fusion and signaling of tumors, among others [2]. The CD63 gene, which is located on human chromosome 12q13, was the first tetraspanin to be characterized [3]. Recent studies have demonstrated that CD63 interacts with many different proteins, either directly or indirectly, and regulates intracellular transport and localization [4,5]. In addition, an increasing number of studies have indicated that the cell surface expression of CD63 is tightly regulated by glycosylation [6]. In fact, the molecular weight of CD63 has been observed to be 32, 35, or 50 kDa with N-linked glycosylation in western blotting experiments, although the predicted molecular weight of CD63 is 25 kDa [7]. Furthermore, it has been reported that CD63 is associated with the biological behavior of solid tumors, especially those with metastatic potential [8]. However, the contribution of glycosylation of CD63 to cancer malignancy is poorly understood.

Previously, we established that glycosylation in multidrug resistance protein 1 (MDR1, also known as ABCB1) is regulated by ribophorin II (RPN2), which is part of an N-oligosaccharyl transferase complex [9]. RPN2 silencing induced docetaxel-dependent apoptosis and cell growth inhibition of human breast cancer cells through the reduction of P-glycoprotein glycosylation. In addition, *in vivo* delivery of RPN2 siRNA inhibited tumor growth in mice given docetaxel. These observations indicated that RPN2 is a key regulator of N-glycosylation in drug-resistant cancer cells. However, little is currently known regarding the association between RPN2 and specific glycosylated proteins that are related to cancer malignancy. In this study, we demonstrate that RPN2 promotes cancer cell malignancy in breast cancer cells through the regulation of CD63 glycosylation.

## Results

### Inhibition of RPN2 expression led to the deregulation of CD63 glycosylation

**Figure 1:**
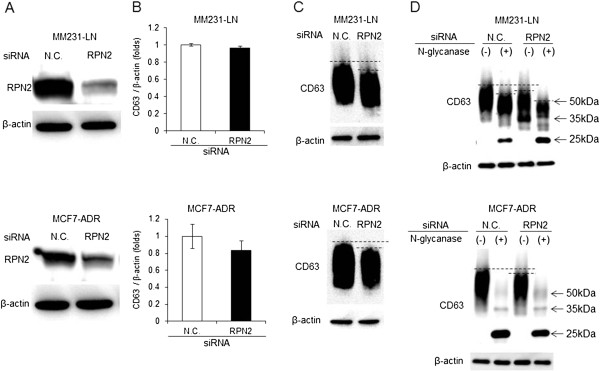
**CD63 glycosylation in breast cancer cells. A)** MDA-MB-231-luc-D3H2LN (MM231-LN) (upper panel) and MCF7-ADR cells (lower panel) were transiently transfected with control (N.C.) or RPN2 siRNAs. After 2 days in culture, RPN2 expression was detected using immunoblotting. β-actin was used as a loading control. **B)** MM231-LN (upper panel) and MCF7-ADR cells (lower panel) were transiently transfected with the N.C. or RPN2 siRNAs. After 2 days in culture, the cell extracts were subjected to qRT-PCR. The values on the y-axis are plotted relative to the expression level of N.C., which is defined as 1. **C)** MM231-LN (upper panel) and MCF7-ADR cells (lower panel) were transiently transfected with N.C. or RPN2 siRNAs. After 2 days in culture, CD63 expression was detected using immunoblotting. β-actin was used as a loading control. Dashed lines show the difference between glycosylated CD63 in the presence or absence of RPN2 siRNA treatment. **D)** Whole cell lysates were collected from MM231-LN (upper panel) and MCF7-ADR cells (lower panel) that were transiently transfected with N.C. or RPN2 siRNA and treated with PBS for 6 hours at 37°C. N.C. and RPN2 siRNA-transfected cells were treated with N-glycosidase for 6 hours at 37°C. CD63 glycosylation was detected by immunoblotting. β-actin was used as a loading control. The CD63 molecular weights of 25 (non-glycosylated), 35 (lower-glycosylated) and 50 kDa (higher-glycosylated) are indicated with arrows to the right.

To investigate whether CD63 was glycosylated by RPN2, MCF7-ADR and MDA-MB-231-luc-D3H2LN (MM231-LN) cells were transiently transfected with siRNA against RPN2, and the glycosylation state of CD63 was examined using western blotting. The reduction in RPN2 expression after transduction with the RPN2 siRNA was confirmed using western blotting (Figure 1A). The RPN2 siRNA had no effect on total CD63 expression in either breast cancer cell line (Figure 1B). However, as shown in Figure 1C, the molecular weight of CD63 decreased in RPN2 siRNA-treated cells compared to control siRNA-treated cells (N.C.) in the MM231-LN (upper panel) and MCF7-ADR (lower panel) cell lines. In addition, to confirm whether the molecular weight of CD63 actually decreased after deglycosylation, N-glycanase was added to cell lysates of MCF7-ADR and MM231-LN cells transfected with control or RPN2 siRNAs. As shown in Figure 1D, the molecular weight of glycosylated CD63 decreased after treatment with N-glycanase in both breast cancer cell lines, suggesting that the smeared band represents the glycosylated form of CD63. Furthermore, a non-glycosylated form of CD63 (25 kDa) and a less glycosylated form of CD63 (35 kDa) emerged from the 50 kDa glycosylated form of CD63 (Figure 1D) [7]. The N-glycanase experiment demonstrated the differences in the molecular weight of various forms of the CD63 protein. These results indicated that RPN2 contributes to the N-glycosylation of CD63 in human breast cancer cells.

### CD63 localization was regulated by RPN2

**Figure 2:**
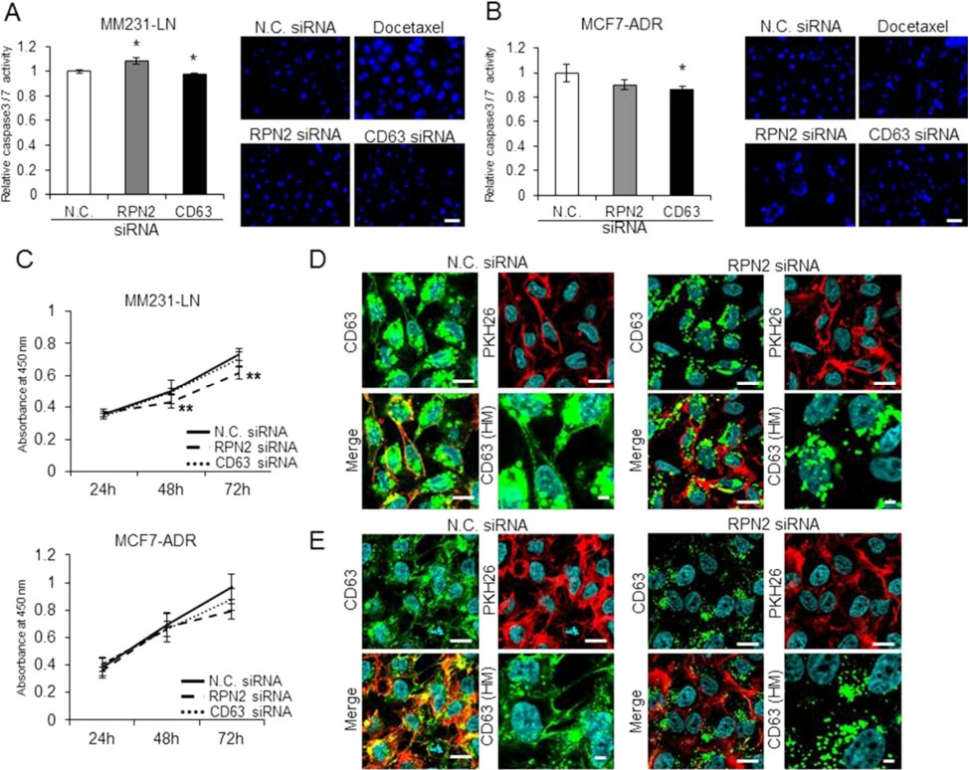
**CD63 localization in breast cancer cells. A)** MDA-MB-231-luc-D3H2LN (MM231-LN) cells were transiently transfected with control (N.C.), RPN2 or CD63 siRNAs. After 2 days in culture, caspase-3/7 activity was assessed using an Apo-ONE Homogeneous Caspase-3/7 Assay system (left panel). The right panels show a representative image of Hoechst 33258-stained sections after N.C., RPN2 or CD63 siRNA transfection. The size bar indicates 50 μm. **B)** MCF7-ADR cells were transiently transfected with control (N.C.), RPN2 or CD63 siRNAs. After 2 days in culture, caspase-3/7 activity was assessed using an Apo-ONE Homogeneous Caspase-3/7 Assay system (left panel). The right panels show a representative image of Hoechst 33258-stained sections after N.C., RPN2 or CD63 siRNA transfection. The size bar indicates 50 μm. **C)** MM231-LN (upper panel) and MCF7-ADR cells (lower panel) were transiently transfected with N.C., RPN2 or CD63 siRNAs. After 1–3 days in culture, cell growth was assessed by an MTS assay. **D)** MM231-LN cells were grown and transiently transfected with N.C. (left panels) or RPN2 siRNA (right panels). After 2 days in culture, immunofluorescence of CD63 (Green) was detected. The blue color indicates nuclei. Cell membranes were stained PKH26 (Red). A higher magnification view is shown (CD63 HM). The size bar indicates 20 μm. **E)** MCF7-ADR cells were grown and transiently transfected with N.C. (left panels) or RPN2 siRNA (right panels). After 2 days in culture, immunofluorescence of CD63 (green) was detected. The blue color indicates nuclei. Cell membranes were stained PKH26 (red). A higher magnification view is shown (CD63 HM). The size bar indicates 20 μm. All data are shown as the mean  S.D., *P<0.05, **P<0.01.

It is well known that glycosylation affects the localization of proteins within the cytoplasm, membranes and pericellular matrix [10]. CD63 is a ubiquitously expressed protein that is localized within the endosomal system [5]. We performed an apoptosis assay using Hoechst staining and a caspase-3/7 assay in the MCF7-ADR and MM231-LN cells after RPN2 or CD63 siRNA transfection. As shown in Figure 2A and B, we found that neither RPN2 nor CD63 silencing induced apoptosis. In addition, knockdown of RPN2 or CD63 slightly inhibited cell proliferation in MCF7-ADR and MM231-LN cells (Figure 2C). To determine the localization of CD63, we performed immunofluorescence staining of CD63 in MCF7-ADR and MM231-LN cells after a control or RPN2 siRNA transfection. In MM231-LN and MCF7-ADR cells transfected with control siRNAs, CD63 was localized in the cell membrane, as indicated by PKH26 staining (Figure 2D: MM231-LN and 2E: MCF7-ADR; N.C.). Notably, CD63 aggregated at the nuclear periphery in RPN2-silenced cells more than in control siRNA treated cells (Figure 2D: MM231-LN and 2E: MCF7-ADR; RPN2 siRNA). These results indicate that the localization of CD63 was regulated by RPN2-mediated N-glycosylation.

### The invasive ability and drug resistance of breast cancer cell lines were regulated by CD63

**Figure 3:**
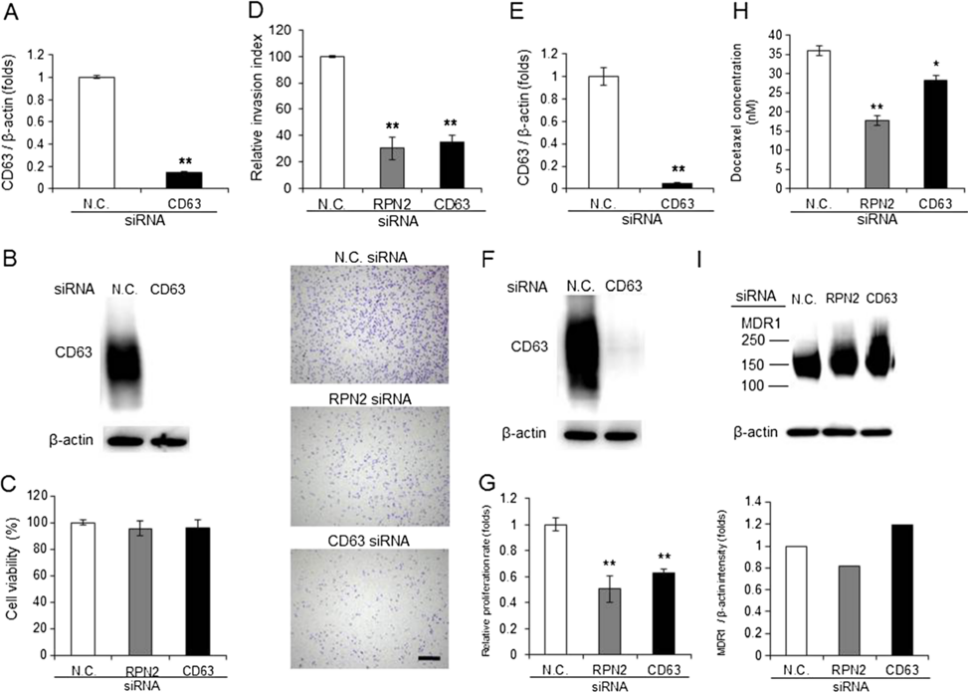
**The association between cancer malignancy and CD63 in breast cancer cells.** MDA-MB-231-luc-D3H2LN (MM231-LN) cells were transiently transfected with the control (N.C.), CD63 or RPN2 siRNAs **(A-D)**. MCF7-ADR cells were transiently transfected with N.C., CD63 or RPN2 siRNAs **(E-I)**. **A)** The cell extracts were subjected to qRT-PCR. The values on the y-axis are plotted relative to the expression level of control (N.C.), which is defined as 1. All data are shown as the mean   S.D., **P < 0.01. **B)** CD63 expression was detected using immunoblotting. β-actin was used as a loading control. **C)** Cell growth assessed by MTS assay. **D)** The following day, the cells were subjected to a Matrigel™ invasion assay. Quantification and representative photographs (lower figure) are shown. The size bar indicates 500  μm. All data are shown as the mean   S.D., **P < 0.01. **E)** The cell extracts were subjected to qRT-PCR. The values on the y-axis are plotted relative to the expression level of N.C., which is defined as 1. All data are shown as the mean   S.D., **P < 0.01. **F)** CD63 expression was detected using immunoblotting. **G)** The medium was replaced with fresh medium containing 25 nM of the chemotherapeutic agent docetaxel for an additional 72 hours. The effect of docetaxel pretreatment on cell viability was examined using an MTS assay. The values on the y-axis are plotted relative to the absorbance of N.C., which is defined as 1. All data are shown as the mean   S.D., **P < 0.01. **H)** The IC50 of MCF7-ADR cells for docetaxel was assessed by MTS assay after transient transfection with the N.C., RPN2 or CD63 siRNAs. All data are shown as the mean   S.D., *P < 0.05, **P < 0.01. **I)** MDR1 expression was detected using immunoblotting. The dashed lines show the molecular weight.

The relationship between CD63 and cancer cell malignancy has previously been reported; however, the exact contribution of CD63 to cancer malignancy is poorly understood. As shown in Figure 2, we found that knockdown of RPN2 led to abnormal localization of CD63, suggesting that disruption of RPN2 expression resulted in the suppression of CD63 function. We previously showed that RPN2 contributes to invasiveness [11] and drug resistance in cancer cells [9]. These observations prompted us to determine whether CD63 contributed to cancer invasiveness or drug resistance. The reduction of CD63 expression after transfection with the CD63 siRNA was confirmed using qRT-PCR and western blotting in MM231-LN cells (Figure 3A and B). The CD63 siRNA had no effect on cell viability 24 hours after transfection in MM231-LN cells (Figure 3C). To examine whether CD63 could influence the invasive ability of breast cancer cells, MM231-LN cells were used in *in vitro* transwell invasion assays after CD63 siRNA treatment. As shown in Figure 3D, the invasion of MM231-LN cells was suppressed by CD63 siRNA treatment compared with the control siRNA treatment. Similarly, inhibition of RPN2 expression by siRNA significantly affected the invasiveness of MM231-LN cells (Figure 3D).Next, the reduction of CD63 expression after transfection with the CD63 siRNA was confirmed using qRT-PCR and western blotting in MCF7-ADR cells (Figure 3E and F). To examine the contribution of CD63 to drug resistance, we measured the half-maximal inhibitory concentration (IC50) of docetaxel in MCF7-ADR cells. We found that CD63 silencing in MCF7-ADR cells (P < 0.05, IC50 = 28.28 ± 1.30 nM) decreased drug resistance against docetaxcel compared to control (IC50 = 35.94 ± 4.42 nM) and RPN2 knockdown cells (P < 0.01, IC50 = 17.67 ± 5.38 nM) (Figure 3G and H). Furthermore, neither CD63 nor RPN2 silencing inhibited MDR-1 expression in MCF7-ADR cells (Figure 3I). Taken together, these results indicated that deregulation of CD63 attenuated drug resistance as well as invasiveness in breast cancer cells. Therefore, the localization of CD63, which is regulated by RPN2, might contribute to cancer malignancy.

### CD63 was co-localized with MDR1 at the cell membrane

**Figure 4:**
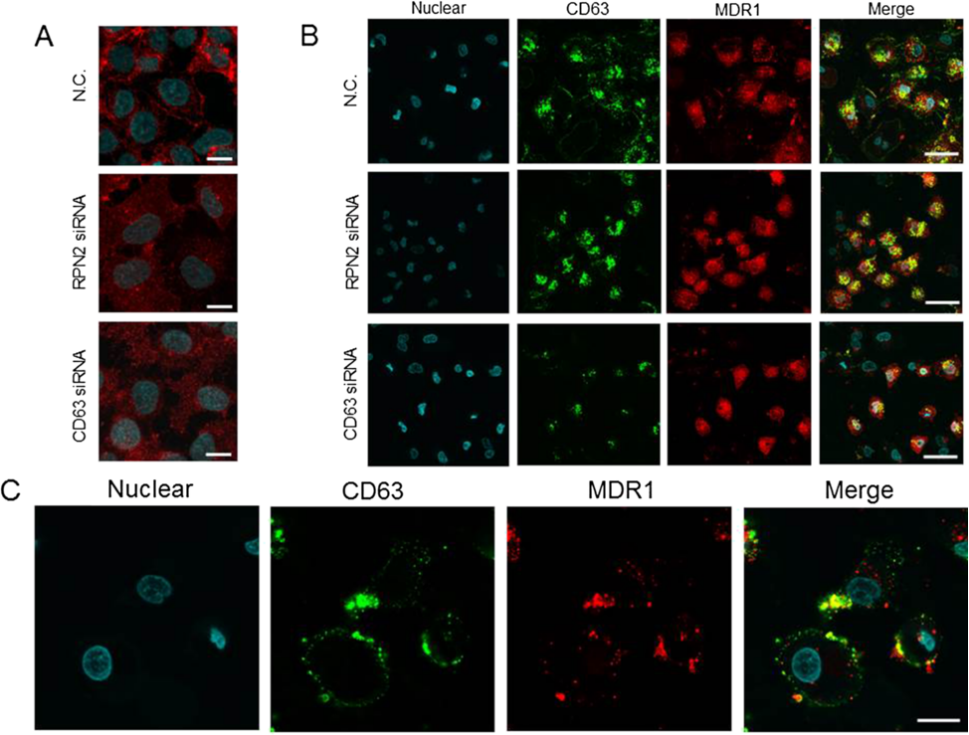
**Effect of RPN2 or CD63 knockdown on MDR1 localization in breast cancer cells. A)** MCF7-ADR cells were grown and transiently transfected with N.C. (upper panel), RPN2 (center panel) or CD63 siRNAs (right panel). After 2 days in culture, the immunofluorescence of MDR1 (Red) was detected. The blue color indicates nuclei. The size bar indicates 20 μm. **B)** MCF7-ADR cells were grown and transiently transfected with the Control (N.C., upper panels), RPN2 (meddle panels) or CD63 siRNAs (lower panels). After 2 days in culture, immunofluorescence of CD63 (Green) and MDR1 (Red) was performed. The blue color indicates nuclei. Co-localization of CD63 and MDR1-positive cells appears as yellow in the overlay image. The size bar indicates 50 μm. **C)** MCF7-ADR cells were grown and transiently transfected with N.C.. After 2 days in culture, the immunofluorescence of CD63 (Green) and MDR1 (Red) was detected. The blue color indicates nuclei. Co-localization of CD63 and MDR1-positive cells appears as yellow in the overlay image. The size bar indicates 20 μm.

We demonstrated that the localization of CD63 was deregulated in MCF7-ADR cells treated with RPN2 siRNA (Figure 2). Previously, we reported that RPN2-knockdown cells showed reduced docetaxel resistance through the reduction of MDR1 membrane localization [9]. As shown in Figure 3C, CD63 siRNA-treated MCF7-ADR cells exhibited increased sensitivity to docetaxel, similar to cells treated with RPN2 siRNA. These results prompted us to hypothesize that the localization of MDR1 is regulated by CD63. To test this hypothesis, immunofluorescence staining was performed using antibodies against CD63 in MCF7-ADR cells. The reduction of the CD63 protein after transduction with siRNA against CD63 was confirmed (Figure 3F). As shown in Figure 4A, the intensity of membrane-bound MDR1 was reduced in MCF7-ADR cells treated with both RPN2 and CD63 siRNAs.

Next, to clarify whether MDR1 localization is regulated by CD63, we conducted a co-immunofluorescence analysis of MCF7-ADR cells treated with control, RPN2 or CD63 siRNAs, using the anti-CD63 and anti-MDR antibodies. Co-immunofluorescence of MDR1 and CD63 indicated that MDR1 was localized at the cell membrane in MCF7-ADR cells transduced with control siRNA, whereas MDR1 was localized in the cytoplasm in RPN2- and CD63-knockdown cells (Figure 4B). Moreover, CD63 co-localized with MDR1 at the cell membrane in MCF7-ADR cells (Figure 4C). Given previous reports showing that CD63 can regulate intracellular and surface trafficking [5], these findings indicated that MDR1 localization was determined by RPN2-mediated glycosylation of CD63. Furthermore, this localization was essential to drug resistance of MCF7-ADR cells.

### Lymph node metastasis was associated with CD63 and MDR1 co-expression in clinical samples

**Figure 5:**
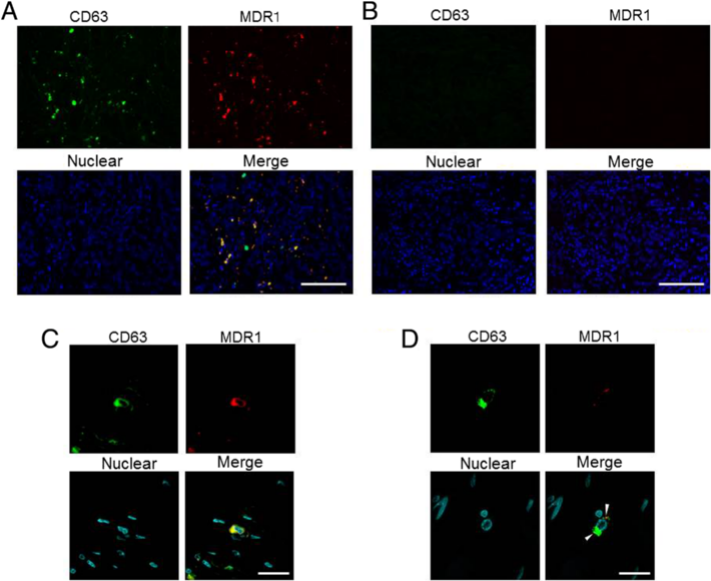
**Co-localization of CD63 and MDR1 in clinical samples.** Tissue microarrays were immunofluorescently stained for CD63 (Green) and MDR1 (Red), as described in the Materials and Methods section. **A)** A representative image of breast cancer tissue that was positive for CD63 and MDR1. **B)** Representative image of breast cancer tissue that was negative for CD63 and MDR1. **C, D)** Co-localization of CD63 and MDR1 (white arrows) was confirmed. Co-localization of CD63 and MDR1-positive cells appears yellow in the overlay image. The blue color indicates nuclei. **A, B)** The size bar indicates 100 μm. **C, D)** The size bar indicates 20 μm.

An association between cancer malignancy and the co-localization of MDR1 and CD63 in breast cancer clinical samples has not been reported. To determine the relationship between CD63 and MDR1 in clinical samples, we performed immunofluorescent staining of CD63 and MDR1 in a 69 breast cancer tissue microarray. Representative results of positive CD63 and MDR1 co-localization are shown in Figure 5A. Figure 5B shows a negative result for CD63 and MDR1 co-localization. A chi-square test of clinicopathological parameters showed that CD63 (P < 0.05) and MDR1 (P < 0.05) were significantly associated with lymph node (LN) metastasis, which was observed in 5 of 8 (62.5%) of the CD63-positive tumors and 12 of 61 (19.7%) of the CD63-negative tumors. In addition, LN metastasis was observed in 3 of 4 (75%) MDR1-positive tumors and 14 of 65 (21.5%) MDR1-negative tumors. These results indicated a positive correlation between CD63 and MDR1 expression and LN metastasis (Table 1).

**Table 1:** The association between cancer malignancy and co-localization of MDR1 and CD63 in breast cancer clinical samples

**LN metastasis**					
		**Number of patients, *n *** **(%)**	**Positive, *n***	**Negative, *n***	**P-value**
**CD63**	Positive	8 (11.6)	5	3	
	Negative	61 (88.4)	12	49	P < 0.05
**MDR1**	Positive	4 (5.8)	3	1	
	Negative	65 (94.2)	14	51	P < 0.05

LN, Lymph node; MDR1, multidrug resistance protein 1.

Next, we performed a co-immunofluorescence analysis in breast cancers and their matched, LN metastatic carcinomas from 48 patients to clarify whether CD63 and MDR1 co-localization is associated with LN metastasis. Among these samples, CD63 co-localized with MDR1 on the cell membrane (Figure 5C and D). In the primary region, CD63-positive staining was observed in 12 of 13 (92.3%) of the MDR1-positive tumors and 12 of 35 (34.3%) of the MDR1-negative tumors (Table 2). In the LN metastatic region, CD63-positive staining was observed in 11 of 11 (100%) MDR1-positive tumors and 16 of 37 (43.2%) MDR1-negative tumors (Table 3). These findings indicated that MDR1 localization was associated with co-localization of CD63 in clinical samples.

**Table 2:** The association between CD63 and MDR1 in breast cancer primary region

**Breast**					
			**CD63**		
		**Number of patients, *n *** **(%)**	**Positive, *n***	**Negative, *n***	**P-value**
**MDR1**	Positive	13 (27.1)	12	1	
	Negative	35 (72.9)	12	23	P < 0.05

MDR1, multidrug resistance protein 1.

**Table 3:** The association between CD63 and MDR1 in breast cancer LN metastatic region

**LN**					
			**CD63**		
		**Number of patients, *n *** **(%)**	**Positive, *n***	**Negative, *n***	**P-value**
**MDR1**	Positive	11 (22.9)	11	0	
	Negative	37 (77.1)	16	21	P < 0.05

LN, Lymph node; MDR1, multidrug resistance protein 1.

## Discussion

Previous studies have shown that the localization of MDR1 at the cell membrane is important to drug resistance in cancer cells [12,13]. Thus, targeting of MDR1 by small molecule compounds or antibodies is an effective strategy for overcoming multiple-drug resistance in cancer [14]. The ultimate goal of restoring drug sensitivity has been met with limited success in clinical trials thus far, although promising studies on the pharmacological inhibition of MDR1 have indicated that it is possible to sensitize drug-resistant cells. Recently, we determined that RPN2 efficiently induced apoptosis in docetaxel-resistant human breast cancer cells [9]. In addition, silencing of RPN2 reduced the glycosylation of MDR1 and decreased its membrane localization, thereby sensitizing cancer cells to docetaxel. Our current results indicate that CD63 glycosylation by RPN2 is important for the localization of CD63 and MDR1 in human breast cancer cells. Indeed, Yoshida et al. showed that CD63 had three N-linked glycosylation sites [15], that CD63 interacts with CXCR4 through the N-linked glycans-portion of the CD63 protein and that the complex induces the direction of CXCR4 trafficking to the endosomes/lysosomes, rather than to the plasma membrane. Moreover, several reports have shown that CD63 interacts with many different proteins, including integrins and the Src family tyrosine kinases Lyn and Hck, which are known to promote cancer malignancy [16-18]. In addition, recent studies have shown that multi-drug resistant cancer cells overexpressing MDR1 displayed increased invasive activity and metastatic behavior [19]. These phenotypes were similar to those observed in RPN2-mediated cancer malignancy [11]. Taken together, our results support the possibility that glycosylated CD63 by RPN2 plays an important role in cancer malignancy through the regulation of protein localization.

We revealed that CD63 interacts with MDR1 and regulates the drug resistance and invasiveness involved in cancer cell malignancy. However, a correlation between decreased expression of CD63 and increased malignancy has also been observed in many other tumors. For instance, it has been reported that CD63 is strongly expressed on the cell surface during the early stage of malignant melanoma, but this localization is weaker or absent in the malignant stages of melanoma compared to normal melanocytes [8]. By contrast, Huang et al. reported that nearly all breast cancers were positive for CD63 mRNA expression [20]. As shown in this study, we found that CD63 localization regulated by RPN2-mediated glycosylation is important for malignant characteristics, including drug resistance and invasiveness. Therefore, to evaluate whether CD63 was associated with cancer malignancy, it was essential to evaluate both the glycosylation and expression level of CD63. Furthermore, we demonstrated that CD63 and MDR1 co-localization was associated with LN metastasis in clinical samples, which suggested that co-localization of CD63 and MDR1 is likely to be a cause for treatment resistance in breast cancer patients.

## Conclusions

**Figure 6:**
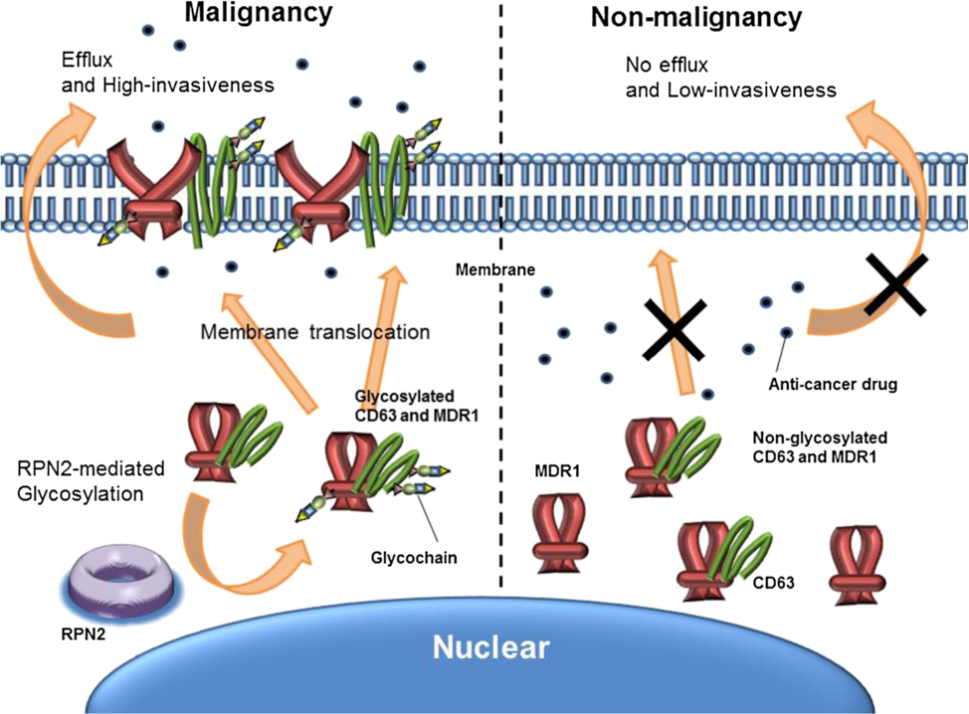
**Schematic model for the mechanism of RPN2-mediated CD63/MDR1 glycosylation pathway in breast cancer cells.** In breast cancer cells, high expression of RPN2 resulted in increased glycosylation of CD63/MDR1 and enhanced membrane localization of CD63/MDR1, which promoted cancer malignancy. In contrast, inhibition of RPN2 expression reduced glycosylation of CD63/MDR1 and decreased membrane localization, thereby attenuating cancer malignancy in breast cancer cells.

In conclusion, this study provides evidence of a novel and important function of RPN2-mediated glycosylation of CD63 in the regulation of MDR1 localization and cancer malignancy, including drug resistance and invasiveness (Figure 6).

## Methods

### Reagents

The antibiotic solution (containing 10,000 U/mL penicillin and 10 mg/mL streptomycin), trypsin-EDTA mixture (containing 0.05% trypsin and EDTA), FBS (fetal bovine serum), donkey anti-mouse-Alexa 488, goat anti-rabbit-Alexa 488, and goat anti-mouse-Alexa 594 were obtained from Invitrogen (Carlsbad, CA, USA). Rabbit polyclonal anti-MDR (H-241, sc-8313) was purchased from Santa Cruz Biotechnology (Santa Cruz, CA, USA). Mouse monoclonal anti-actin, clone C4 (MAB1501) was purchased from Millipore (Billerica, MA, USA). The mouse monoclonal anti-CD63 monoclonal (H5C6) antibody was obtained from BD Pharmingen (San Diego, CA, USA). Hoechst 33258 dye was obtained from Dojindo (Kumamoto, Japan). Antigen activation of the tissue microarray was achieved using a protease (#415231, Nichirei, Japan). The duplexes of each siRNA targeting human CD63 mRNA (si CD63-1, GGUGGAAGGAGGAAUGAAAdTdT, UUUCAUUCCUCCUUCCACCdTdT; CD63-2, GGCAGCAGAUGGAGAAUUAdTdT, UAAUUCUCCAUCUGCUGCCdTdT; CD63-3, GUGGCUACGAGGUGAUGUAdTdT, UACAUCACCUCGUAGCCACdTdT) were purchased from BONAC Corporation (Fukuoka, Japan). The siRNA duplexes targeting human RPN2 mRNA (GGCCACUGUUAAACUAGAACA, UUCUAGUUUAACAGUGGCCUG) were purchased from Sigma-Aldrich (St. Louis, MO, USA), and the AllStars Negative Control siRNA was obtained from Qiagen (Valencia, CA, USA).

### Cell culture

MDA-MB-231-luc-D3H2LN (MM231-LN) cells were purchased from Xenogen (Alameda, CA), and multidrug-resistant MCF7-ADR cells were provided by Shien-Lab, Medical Oncology, National Cancer Center Hospital of Japan. These cells were maintained in RPMI 1640 medium (Invitrogen, Carlsbad, CA, USA) supplemented with 10% heat-inactivated FBS and antibiotic-antimycotic at 37°C in 5% CO^2^.

### Transient transfection assays

Transfection of siRNA was accomplished using DharmaFECT transfection reagent (Thermo Scientific, Waltham, MA, USA) according to the manufacturer’s protocol. AllStars Negative Control siRNA was used as the negative control (N.C.).

### Cell proliferation assay (MTS assay)

Five thousand cells per well were seeded in 96-well plates. The following day, the cells were transfected with siRNAs. After 1, 2 or 3 days of culture, cell viability was measured using a cell counting Kit-8 (Dojindo, Kumamoto, Japan) according to the instructions of the manufacturer. The absorbance at 450 nm was measured using an Envision multilabel plate reader (Wallac, Turku, Finland).

### Apoptotic activity

MM231-LN and MCF7-ADR cells (2 × 10^5^ cells in a 6-well plate) were transfected with control, RPN2 or CD63 siRNA as described above. After 2 days in culture, cells were collected, and proteins were extracted with M-PER (Thermo Scientific). Caspase-3/7 activity was assessed using an Apo-ONE Homogeneous Caspase-3/7 Assay system (Promega, Wisconsin, USA) at an excitation wavelength of 480 nm and an emission wavelength of 520 nm using an Envision system (Wallac).

### Transwell invasion assay

Breast cancer cell invasion was assayed in 24-well Biocoat Matrigel™ invasion chambers (8 μm; BD Pharmingen, San Diego, CA, USA) according to the manufacturer’s protocol. Briefly, after the transfection of siRNA into the cells, 20,000 cells were plated in the upper chamber containing RPMI 1640 medium without FBS on the following day. The lower chambers were filled with RPMI 1640 medium with 10% FBS as a chemoattractant. Twenty-two hours later, the low-invasive cells were removed with a cotton swab. The cells that migrated through the membrane and adhered to the lower surface of the membrane were fixed with methanol and stained with Diff Quick staining solution (Sysmex, Kobe, Japan). For quantification, the cells in four random fields were counted using a microscope. All assays were performed in triplicate, and the invasive values were normalized to the values from cells transfected with the AllStars Negative Control siRNA.

### Isolation of mRNAs and quantitative real-time PCR (qRT-PCR)

Total RNA was extracted from cultured cells using a miRNeasy Mini Kit (Qiagen, Valencia, CA, USA) according to the manufacturer’s protocol. The qRT-PCR method has previously been described [21]. PCR was performed in 96-well plates using a 7300 Real-Time PCR System (Applied Biosystems, Foster City, CA, USA), and all reactions were performed in triplicate. TaqMan® qRT-PCR kits and human-CD63 and human-β-actin TaqMan® Expression Assays were purchased from Applied Biosystems (Foster City, CA, USA). Reverse transcription (Applied Biosystems, Foster City, CA, USA) and TaqMan® quantitative PCR (Applied Biosystems, Foster City, CA, USA) were performed according to the manufacturer’s instructions. SYBR® Green I qRT-PCR was performed, and the β-actin housekeeping gene was used to normalize the variation in the cDNA levels. The primer sequences are as follows (shown 5′ to 3′): human β-actin, GGCACCACCATGTACCCTG (Forward) and CACGGAGTACTTGCGCTCAG (Reverse); and human RPN2, ATCTAACCTTGATCCCAGCAATUGTG (Forward) and CTGCCAGAAGCAGATCTTTGGTC (Reverse).

### Immunoblot analysis

SDS-PAGE gels were calibrated using Precision Plus protein standards (161–0375) (Bio-Rad, Hercules, CA, USA), and anti-CD63 (1:200) and anti-actin (1:1,000) were used as the primary antibodies. The dilution ratio of each antibody is indicated in parentheses. A peroxidase-labeled anti-mouse secondary antibody was used at a dilution of 1:10,000. The bound antibodies were visualized using chemiluminescence with an ECL Plus Western blotting detection system (GE HealthCare, Piscataway, NJ, USA), and luminescent images were captured using a LuminoImager (LAS-3000; FujiFilm Inc., Tokyo, Japan).

### Immunofluorescent staining

After being washed three times with PBS, the cells were fixed in 4% paraformaldehyde (Wako, Japan) and incubated in 0.1% BSA containing primary antibodies (anti-CD63 (1:500) and anti-MDR (1:500) for 1 hour. The cells were then incubated in 0.1% BSA containing Alexa Fluor fluorescent secondary antibodies. Nuclei were visualized with Hoechst 33258 dye (Dojindo, Kumamoto, Japan). All staining was observed using a confocal microscope (FluoView FV1000; Olympus, Tokyo, Japan).

### Cell membrane labeling

MDA-MB-231-luc-D3H2LN and MCF7-ADR cells were transfected with control or RPN2 siRNA. After 2 days in culture, cells were labeled with a PKH26 red fluorescent labeling kit (Sigma Aldrich). Cells were observed using confocal microscopy (FluoView FV1000; Olympus, Tokyo, Japan). Nuclei were visualized via Hoechst 33258 (Dojindo, Kumamoto, Japan) staining.

### Tissue microarrays

The tissue arrays of breast cancer samples (BR1503b, BR10010a) were purchased from Biomax US. The company provided certified documents that all human tissue samples were collected with informed consent from the donors or their relatives. Detailed information on all tumor samples can be found at http://www.biomax.us/. The tissue microarrays were incubated in 0.1% BSA containing primary antibodies, including anti-CD63 (1:500) and anti-MDR (1:500), for 1 hour after a 5-min protease treatment (Nichirei, Tokyo, Japan). The cells were then incubated in 0.1% BSA containing Alexa Fluor fluorescent secondary antibodies. Nuclei were visualized using Hoechst 33258 (Dojindo, Kumamoto, Japan) staining for observation using a confocal microscope (BZ-9000; Keyence, Tokyo, Japan).

### Statistical analysis

The data presented in bar graphs are the mean and s.e.m. of at least three independent experiments. Statistical analyses were performed using Student’s t-test. Associations between lymph node metastasis or MDR expression and CD63 expression were assessed by means of a chi-square test. The statistical analysis was two-sided, and P < 0.05 was considered to be significant.

## Competing interests

The authors declare that they have no competing interests.

## Authors’ contributions

NT and KH performed the experimental work, conducted data analysis and wrote the manuscript. NT’s main contribution was obtaining proof of the relationship between CD63 and anti-cancer drug resistance. KH obtained evidence of the relationship between CD63 and invasiveness. NK assisted in the writing of the manuscript and provided helpful discussion. HN provided helpful discussion. TO supervised this project. The manuscript was finalized by TO with the assistance of all of the authors. All authors read and approved the final manuscript.
